# Neuromodulatory effects of offline low-frequency repetitive transcranial magnetic stimulation of the motor cortex: A functional magnetic resonance imaging study

**DOI:** 10.1038/srep36058

**Published:** 2016-10-27

**Authors:** Yu-Sun Min, Jang Woo Park, Seong Uk Jin, Kyung Eun Jang, Byung-Joo Lee, Hui Joong Lee, Jongmin Lee, Yang-Soo Lee, Yongmin Chang, Tae-Du Jung

**Affiliations:** 1 Department of Physical Medicine and Rehabilitation, Kyungpook National University Hospital, Korea; 2 Department of Biomedical Engineering, Seoul National University College of Medicine, Korea; 3 Department of Medical & Biological Engineering, Kyungpook National University, Korea; 4 Department of Radiology, Kyungpook National University Hospital, Korea; 5 Department of Molecular Medicine, Kyungpook National University School of Medicine, Korea

## Abstract

Repetitive transcranial magnetic stimulation (rTMS) of the primary motor cortex (M1) can modulate cortical excitability and is thought to influence activity in other brain areas. In this study, we investigated the anatomical and functional effects of rTMS of M1 and the time course of after-effects from a 1-Hz subthreshold rTMS to M1. Using an “offline” functional magnetic resonance imaging (fMRI)-rTMS paradigm, neural activation was mapped during simple finger movements after 1-Hz rTMS over the left M1 in a within-subjects repeated measurement design, including rTMS and sham stimulation. A significant decrease in the blood oxygen level dependent (BOLD) signal due to right hand motor activity during a simple finger-tapping task was observed in areas remote to the stimulated motor cortex after rTMS stimulation. This decrease in BOLD signal suggests that low frequency subthreshold rTMS may be sufficiently strong to elicit inhibitory modulation of remote brain regions. In addition, the time course patterns of BOLD activity showed this inhibitory modulation was maximal approximately 20 minutes after rTMS stimulation.

Repetitive transcranial magnetic stimulation (rTMS) has received increasing interest as a tool for modulating cortical excitability in a range of clinical settings and experimental conditions^1^. While single pulse TMS can briefly disrupt or excite underlying cortical tissue, rTMS results in changes in cortical excitability beyond the duration of stimulation, thus showing promise as a tool for neurorehabilitation for various neurologic and psychiatric conditions. rTMS can influence ongoing neural processing such that it enhances cortical excitability^2^,^3^. Although rTMS at low frequencies (<5 Hz) is known to decrease cortical excitability^4^ and rTMS at higher frequencies (>5 Hz) increases it^5^, there is still ongoing debate about the level of frequency that promotes neural inhibition and the levels that facilitates neural activity. Recently, neuronavigation guiding of rTMS has increased targeting accuracy to improve the limited spatial resolution of conventional rTMS^6^,^7^.

The primary motor cortex (M1) is the most frequently stimulated cortical site in rTMS studies. Focal rTMS to M1 in humans was reported to influence motor activity in both normal and diseased populations. However, the effect of rTMS on motor task-related neural activity remains controversial. For example, modulation of task-related neural activity in M1 contralateral to the stimulation site has been reported, but both increases^8^,^9^ and decreases^10^ in activity were reported.

Another issue in rTMS research is the time course of after-effects. The time course of after-effects is important because it reflects prolonged synaptic plasticity changes beyond stimulation, and thus provides valuable information regarding the optimal interval between rTMS interventions. Although the effects of rTMS vary depending on stimulation frequency, intensity, duration, and experimental design^11^,^12^,^13^, it remains uncertain how rTMS after-effects affect neural activity across different brain regions over time. Several studies measured the duration of electrophysiological effects resulting from low frequency rTMS of M1 using various methodologies such cortical excitability (single pulse TMS) and cortical inhibition (paired pulse protocols)^14^,^15^,^16^,^17^,^18^. Functional imaging studies also explored the after-effects of TMS on motor cortex^19^,^20^. However, while functional imaging studies have traditionally investigated task-induced activation (increases in brain activity), much interest has recently been focused on task-induced deactivation (decreases in brain activity) that occurs during task performance relative to rest^21^,^22^.

In the current study, we used an “offline” functional magnetic resonance imaging (fMRI)-rTMS paradigm to investigate two questions: (1) whether the effect of rTMS on M1 is limited to brain structures within or beyond the motor network, and (2) whether the time course of the neural activity associated with motor task shows a different activation pattern before and after a low frequency rTMS over M1. First, we investigated and interpreted the inhibitory effects of rTMS on M1 from both the classical motor network perspective and within a multi-network framework. We hypothesized that rTMS of M1 may induce neural modulation of not only sensorimotor networks, but also of non-motor networks during active motor tasks. Second, we investigated the time course of after-effects from rTMS of M1 using a serial consecutive fMRI. This is essential because previous studies often included only a cross-sectional setting, thus results simply reflected differences between pre- and post-rTMS without providing information on the time course of after-effects. To the best of our knowledge, our offline fMRI-rTMS approach illustrates for the first time the extent and time course of rTMS stimulation–induced BOLD activity. For targeting of the M1 across subjects and stimulation sessions, we employed an MRI-guided rTMS method.

## Results

### rTMS

rTMS was well tolerated and no subject reported relevant side effects. Mean resting motor threshold (RMT) was 69.2 ± 7.5%, ranging from 54 to 91% of maximum Magstim rapid stimulator output. Mean Montreal Neurological Institute (MNI) normalized M1 coordinates, stored in the neuronavigation software during rTMS, were −40, −22, and 58, corresponding to the left M1. Individually localized target left M1 coordinates in each subject were summarized in Supplementary Table 1.

### BOLD activity changes during the finger-tapping task pre- and post-rTMS

**Figure 1:**
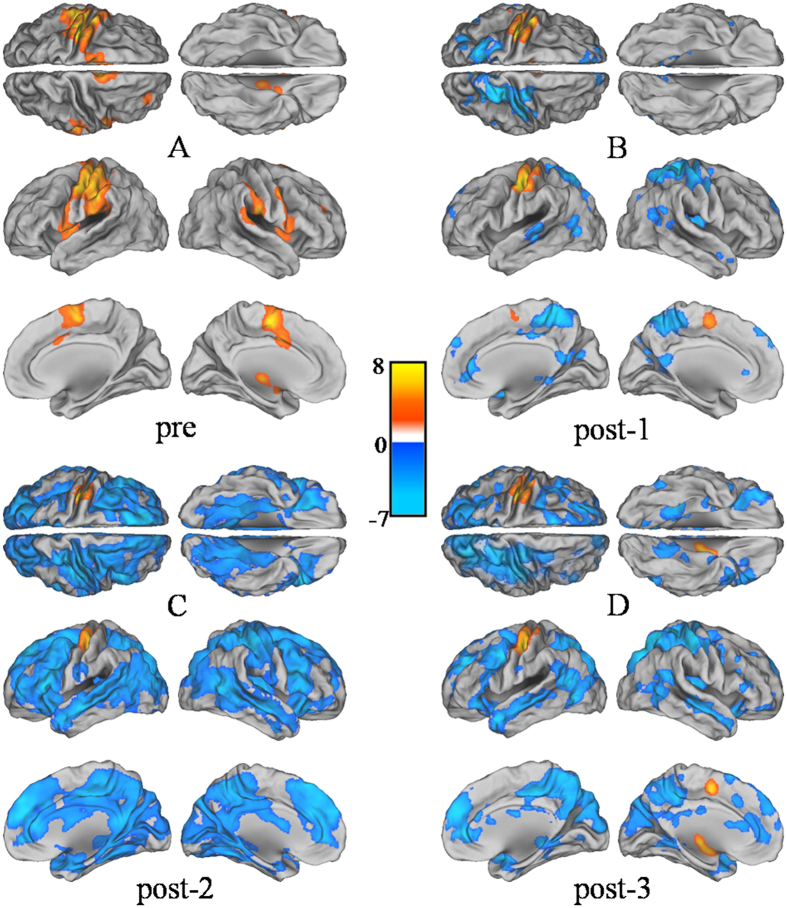
Group one sample t-test results (p < 0.05, FDR-corrected for multiple comparisons at the whole brain level). Activation (motor task > rest) was presented in yellow and deactivation (motor task < rest) was presented in blue. (**A**) pre-rTMS, (**B**) post1, (**C**) post2, and (**D**) post3. After rTMS of the left M1, within group analysis of the right finger-tapping task showed that the extent of motor activation was reduced at post1, post2 and post3, although the activities of ipsilateral motor networks slightly recovered at post3. The most striking effect of rTMS was significant deactivation in sensory and non-motor brain areas. These deactivations were most significant at post2.

Figure 1 shows group-level activation during the finger-tapping task at pre- and three post-rTMS time points. During pre-rTMS, activation (positive BOLD responses) was seen in the left precentral gyrus (M1), left postcentral gyrus (S1), bilateral SMA, left ventral premotor cortex (vPMC) and subcortical regions (left thalamus, putamen, and right cerebellum) when contrasted with baseline using one sample t-testing. (p < 0.05, FDR-corrected at the voxel level). Although it did not reach statistical significance, deactivation (negative BOLD response) was seen in the right M1. During post-rTMS, activation was seen mostly in the left M1, bilateral SMA, and right cerebellum. Statistically significant deactivation appeared in the right S1, bilateral posterior parietal cortices, and bilateral inferior frontal cortex(threshold p < 0.05, FDR-corrected for multiple comparisons across the whole brain).

### BOLD activity changes: One-way ANOVA and post-hoc two-sample analysis

**Figure 2:**
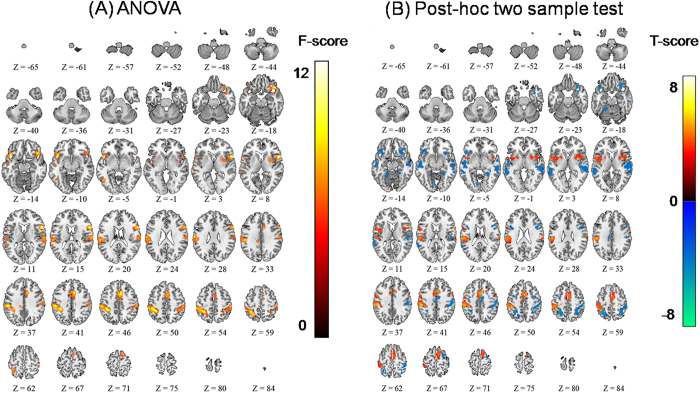
(**A**) One-way within subject ANOVA for the four conditions (pre, post1, post2, and post3). The SPM{F}s had a threshold of p < 0.05, with FDR-correction for multiple comparisons at the whole brain level. (**B**) Post-hoc two sample t-tests showed that the difference between the pre and post2 conditions (pre > post2) was responsible for the difference shown in ANOVA. The SPM{t}s had a threshold of p < 0.05, with a Bonferroni adjusted alpha level of 0.0084, and FDR-correction for multiple comparisons at the whole brain level. Activation differences were presented in yellow and deactivation differences were presented in blue.

One-way ANOVA analysis of the four conditions (pre- and three post-rTMS time points) showed significant BOLD activation changes in the bilateral S1, SMA, bilateral insula, and bilateral inferior frontal region (threshold p < 0.05, FDR-corrected for multiple comparisons across the whole brain, Fig. 2A and Supplementary Table 2). Post-hoc two-sample t-tests were performed for further comparison between conditions. First, contrast comparison for activation showed significant differences between pre- and 20 minutes post-rTMS (post 2) in the left S1, SMA, and bilateral insula (threshold p < 0.05, FDR-corrected for multiple comparisons across the whole brain, Fig. 2B and Supplementary Table 3). This comparison for activation therefore showed that the percentage positive signal change at pre-stimulation was larger than the percentage negative signal change at post 2. Second, contrast comparison for deactivation showed significant differences between pre- and 20 minutes post-rTMS (post 2) in the right S1, temporal cortex, posterior parietal cortex, and inferior frontal cortex bilaterally (threshold p < 0.05, FDR-corrected for multiple comparisons across the whole brain, Fig. 2B and Supplementary Table 3). Similarly, this comparison for deactivation therefore showed that the percentage negative signal change at post 2 was larger than the percentage positive signal change at pre-stimulation.

### Time course of BOLD activity

**Figure 3:**
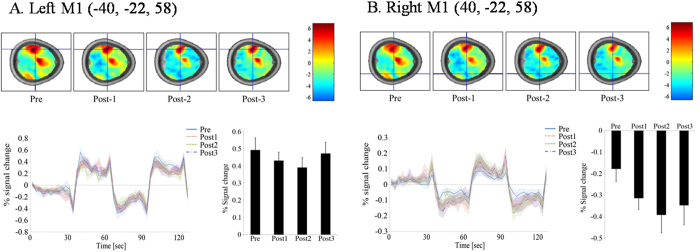
Results of the right finger-tapping task during four BOLD fMRI scans before and after 1-Hz rTMS of the left (**A**) and right M1 (**B**). Group one sample t-test maps had no threshold to show time course of changes in whole brain neural activity. (**A**) A time course of BOLD activity in the left M1 showed positive BOLD signal (activation) during right finger-tapping in four conditions (pre, post1, post2, and post3). The mean percent signal change was slightly reduced at post1 and post2, but returned to the pre-rTMS value. The reduction in percent signal change at post1 and post2 was not statistically significant. (**B**) Time course of BOLD activity in the right M1 showed negative BOLD signal (deactivation) during right finger-tapping at four conditions (pre, post1, post2 and post3). Deactivation was slightly increased at post1, post2, and post3, but there was no statistical significance. Plots showed means (lines) and SEMs (shading) of percentage signal changes.

**Figure 4:**
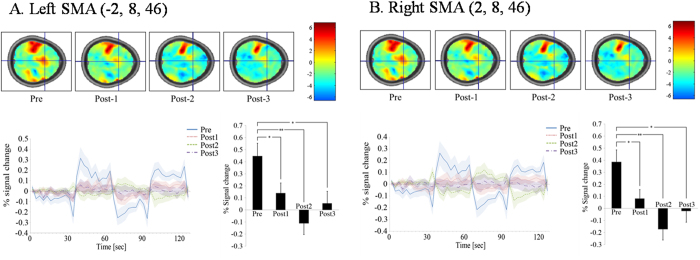
Results of the right finger-tapping task during four BOLD fMRI scans before and after 1-Hz rTMS of the left (**A**) and right SMA (**B**). Group one sample t-test maps had no threshold to show time course of changes in whole brain neural activity. (**A**) A time course of BOLD activity in the left SMA showed changes from positive responses (activation) from pre-stimulation to negative responses (deactivation) at post2, then back to positive responses (activation) at post3. (**B**) Time course of BOLD activity in the right SMA showed changes from positive responses (activation) at pre-stimulation to negative responses (deactivation) at post2 and post3. (*) P < 0.05 and (**) P < 0.01. Plots showed means (lines) and SEMs (shading) of percentage signal changes.

In the left M1 (rTMS stimulation site), the BOLD activity showed positive responses (activation) during right finger-tapping at pre- and post-rTMS time points (Fig. 3A). Although not statistically significant, there was a tendency that activation slightly decreased until 20 minutes after rTMS (post2), and then back to pre-stimulation levels. On the other hand, in the right M1 region, the BOLD activity showed negative responses (deactivation) during right finger-tapping to pre and three post-rTMS time points (Fig. 3B), although statistical significance was not reached. However, in the SMA region, there was a significant decrease in BOLD activation between pre-, post2 (20 minutes after rTMS), and post3 (30 minutes after rTMS) (p < 0.01). That is, in the case of the left SMA, BOLD activity showed changes from positive responses (activation) from pre-stimulation to negative responses (deactivation) at post2, then back to positive responses (activation) at post3 (Fig. 4A). In the case of the right SMA, BOLD activity showed changes from positive responses (activation) at pre-stimulation to negative responses (deactivation) at post2 and post3 (Fig. 4B).

**Figure 5:**
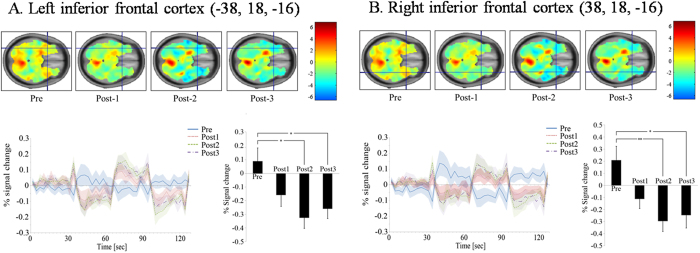
Results of the right finger-tapping task during four BOLD fMRI scans before and after 1-Hz rTMS of the left (**A**) and right inferior frontal cortex (**B**). Group one sample t-test maps had no threshold to show time course of changes in whole brain neural activity. A time course of BOLD activity in the left and right inferior frontal cortex showed changes from positive responses (activation) from pre-stimulation to negative responses (deactivation) at post1, post 2 and post3. (*) P < 0.05 and (**) P < 0.01. Plots showed means (lines) and SEMs (shading) of percentage signal changes.

In the left S1 region, BOLD activity showed changes from positive responses (activation) from pre-stimulation to slightly negative responses (deactivation) at 20 minutes after rTMS (post2), then back to positive responses (activation) (Supplementary Figure 1A). The difference between pre-, post2, and post3 was statistically significant (p < 0.01). In the right S1 region, this time pattern was more prominent (Supplementary Figure 1B). That is, BOLD activity showed statistically significant changes from positive responses (activation) from pre-stimulation to negative responses (deactivation) at post2 and post3 (p < 0.01). Similarly, in the left and right inferior frontal region (Fig. 5A,B), BOLD activity showed statistically significant changes from positive responses (activation) from pre-stimulation to negative responses (deactivation) at post2 and post3 (p < 0.01). The time correlation between time courses of the three post-stimulations and pre-stimulation are summarized in Supplementary Table 4.

### Functional imaging: Sham rTMS

Supplementary Figure 2 shows group-level activation during the finger-tapping task at pre- and three post-sham stimulation time points. During pre-sham stimulation, activation was seen in the left precentral gyrus (M1), left postcentral gyrus (S1), bilateral SMA, and subcortical regions (left thalamus, putamen, and right cerebellum) when contrasted with baseline using one sample t-testing. (p < 0.05, FDR-corrected at the voxel level). During post-sham stimulation, activation was seen mostly in the left M1, left S1, and bilateral SMA. One-way ANOVA analysis of the four conditions (pre- and three sham rTMS time points) showed no significant changes in BOLD activation/deactivation during right finger-tapping (threshold p > 0.05, FDR-corrected for multiple comparisons across the whole brain).

## Discussion

In the present study, we focused on two issues: (1) whether the effect of inhibitory rTMS on M1 is limited to brain structures within the motor network and (2) whether there is a change in the time course pattern of inhibitory rTMS after-effects during an active finger-tapping task. The main findings of this study are (1) inhibitory rTMS of the left M1 induces BOLD activity not at the stimulation site, but in remote brain regions. Specifically, inhibitory rTMS of the left M1 resulted in widespread deactivation of non-motor networks. (2) The time course of inhibitory rTMS after-effects revealed that inhibitory rTMS of the left M1 resulted in widespread deactivation of sensory and non-motor regions. These deactivations were maximal 20 minutes after rTMS. Such after-effects of cortical rTMS have already been reported. However, to the best of our knowledge, our study illustrates the extent and time course of rTMS induced activity suppression for the first time.

The current study showed that 1-Hz rTMS over left M1 at a 90% motor threshold (MT) induced alterations in BOLD activity not at the site of stimulation, but in remote brain regions anatomically and functionally connected with the stimulation site. This finding is in line with previous reports that subthreshold rTMS over M1 induces BOLD signal changes during hand movement only in remote brain regions after stimulation^23^,^24^.

Based on modeling^25^, one possible explanation for no detectable BOLD response changes at the stimulation site is that local rTMS-induced activity changes are less apparent because both excitatory and inhibitory neural populations are affected. Thus, net activation is weak and difficult to detect, whereas longer range connections are mainly excitatory pathways leading to stronger and longer-lasting remote responses that are more easily observed. However, previous PET-TMS studies have reported strong regional cerebral blood flow (rCBF) increases at the site of stimulation during motor activity when using longer stimulation periods (up to 30 minutes)^9^,^26^,^27^. One possibility might be the difference in stimulation time between studies. The absence of a BOLD effect to a brief subthreshold rTMS seems to indicate a dose-dependency that leads to different cortical (and hemodynamic) effects for prolonged stimulation compared to a short series of stimuli.

Subthreshold 1-Hz rTMS induced significant BOLD activity changes in remote brain regions, including the SMA, sensory cortex, and frontal cortices. Specifically, the current study demonstrated that anatomically connected brain regions within motor networks showed reduced activation, but functionally connected brain regions outside motor networks showed strong deactivation. Previous fMRI-rTMS studies also demonstrated deactivation in remote brain regions after TMS stimulation of M1^28^,^29^. Furthermore, our data showed that a unilateral simple finger movement is associated with deactivation of the ipsilateral M1 cortex and deactivation of the sensory cortex of both hemispheres, in addition to the well-known activation of the contralateral M1 cortex. Ipsilateral M1 deactivation could result from transcallosal inhibition, whereas intracortical M1–sensory connections could be responsible for the sensory deactivation. Therefore, our data can also be interpreted to suggest a possibility that other brain areas change their activity to perform a motor behavior when the area that usually performs that behavior (left M1) has been altered by rTMS.

Although activation most likely reflects an increase in excitatory neuronal activity, it is much more difficult to assign deactivation to a particular change in neural activity^30^. For example, using a novel optogenetic fMRI, it was recently demonstrated that specific stimulation of local excitatory neurons in the neocortex or thalamus elicits positive BOLD signals at the stimulus location^31^. However, studies demonstrated a decrease in BOLD signal in healthy subjects over time across multiple sessions of a simple motor task, suggesting habituation or attentional modulation take places^32^,^33^. Our study design, however, excludes the presence of mere habituation or attentional processes by including a control condition (sham stimulation) using the same task, therefore controlling for any putative habituation or attentional modulation. An alternative explanation for deactivation suggests that altered cortical excitability induced by rTMS might change the efficacy of neural signal transmission, resulting in reduced postsynaptic field potentials and BOLD signal. Although the mechanisms by which rTMS exerts these effects are not completely understood, it is quite plausible that rTMS over M1 facilitates remote network efficacy during motor activity by increasing inhibitory activity, therefore decreasing the overall BOLD signal^34^,^35^,^36^,^37^.

In association with deactivation in remote brain regions, another interesting finding in this study is the time course of BOLD responses during motor activity after rTMS. Sensorimotor networks showed hemispheric differences in the time course of BOLD responses during motor activity after rTMS. The sensory cortices in both hemispheres showed the same time course as the left M1 during motor activity before rTMS. After rTMS, the right sensory cortex showed longer lasting deactivation than left sensory cortex, suggesting prolonged inhibitory modulation of the contralateral (right) hemisphere compared to the ipsilateral (left) hemisphere. Therefore, it is tempting to speculate that the strong and long lasting inhibitory modulation of the right sensorimotor network may cause strong suppression of neuronal signals from the right corticospinal pathway associated with right hand activity. On the other hand, brain structures in non-sensorimotor networks showed strong and long lasting deactivation in both hemispheres after rTMS without hemispheric differences. Although previous studies demonstrated rTMS induces regional changes at the stimulation site (e.g., M1) and at spatially remote parts of the brain^30^,^38^,^39^,^40^,^41^, the current study is the first, to our knowledge, demonstrating rTMS over M1 causes significant and long lasting inhibitory modulation of non-motor networks. The possible implication of this finding is that 1-Hz subthreshold rTMS over M1 may be used therapeutically for both motor systems and cognitive systems by modulating functional connectivity between networks. It is clear that more study is required to delineate the precise physical and biological mechanisms leading to these implications. Such work should provide further understanding of the uses of rTMS as a potential treatment tool for neurological diseases, such as cognitive deficit.

This study has limitations. First, motor behavior such as the rate of finger tapping was not measured although participants were instructed to keep the same rate of finger tapping. Therefore, there is uncertainty whether the brain changes occurred to maintain consistent behavior or occurred because behavior itself was altered. Future study is warranted to resolve this uncertainty. Second, although the participants practiced the motor task several times before the fMRI recording session to avoid a possible order effect/training effect, the short-term motor learning/order effect were not fully addressed in this study. Therefore, when interpreting the results, the possibility of order effect should be considered as a potential source for the results found in the current study because such order effect/training effect cannot be completely excluded.

In conclusion, this study demonstrated that 10 minutes of “offline” 1-Hz subthreshold rTMS over M1 leads to task-related BOLD activity changes in remote brain regions, but not at the stimulation site. Furthermore, altered task-related BOLD activity in remote brain regions showed strong deactivation, suggesting low frequency subthreshold rTMS is sufficiently strong to elicit inhibitory modulation of remote brain regions during finger movements. Alternatively, our findings can also be interpreted to demonstrate that remote brain regions change their BOLD activity to compensate for focal distruption from rTMS. The time course patterns of BOLD activity showed that this modulation was maximal ~20 minutes after rTMS stimulation and returned to initial patterns.

## Methods

### Subjects

Twenty healthy, right-handed volunteers (14 men and 6 women) between 23 and 36 years (mean age: 28.6 years) were recruited. Exclusion criteria included a history of brain injury, neurologic or psychiatric disease, any major medical illness, contraindications to TMS^42^, or intake of any medication during the time of study. Written informed consent was obtained from all participants. The study adhered to the Declaration of Helsinki and was approved by the Institutional Review Board from Kyungpook National University Hospital (IRB#: 2014-02-027).

### Experimental design

**Figure 6:**
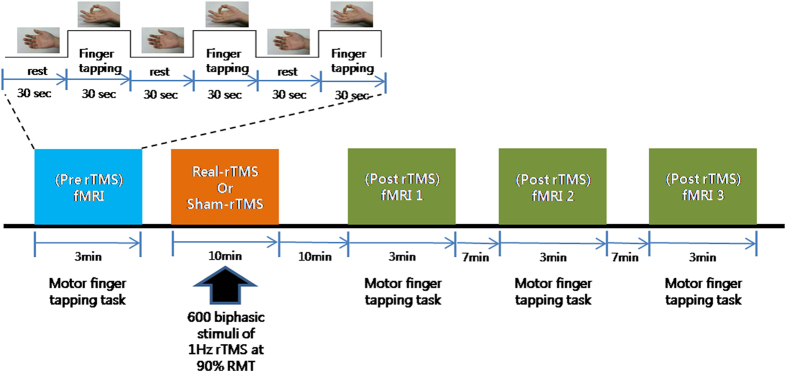
Experimental design. Subjects underwent four BOLD-fMRI scans before and after 1-Hz real- or sham-rTMS. Simple finger-tapping tasks were performed during fMRI. After pre-rTMS fMRI, 600 biphasic stimuli of 1 Hz rTMS were given over the left M1 hand area. All subjects received 10-minute trains of 1-Hz rTMS delivered outside the fMRI scanner. Post-rTMS fMRI was performed three times starting 10 minutes after application of 10-minute trains of 1-Hz rTMS. Real- and sham-rTMS were performed on separate days.

Figure 6 illustrates the study design. The within-subject design was comprised of three sessions: an fMRI session before rTMS stimulation, rTMS session, and an fMRI session after rTMS stimulation. For each fMRI session, six blocks (three active finger-tapping blocks and three resting blocks) were employed. During a finger tapping block, a self-paced simple finger-tapping task using a thumb and an index finger was performed at approximately 1 Hz frequency. In addition, before the fMRI session, the participants had a practice session to familiarize with the experimental procedures including finger-tapping motor task. Participants practiced finger tapping task several times until they performed the task almost automatically. Furthermore, it is expected that the motor practice session may mitigate the potential neural phenomena associated with motor learning. For rTMS, 1-Hz rTMS over the left M1 was performed. Post-rTMS sessions consisted of three consecutive fMRI scans (post1, post2, and post3) with 10-minute intervals. The after-effects of rTMS were assessed by three consecutive fMRI scans after rTMS. For control sessions, the same within-subject design with sham rTMS was employed on different days at least one month apart to wash out the effect of previous real rTMS.

### Location of the target region

Subjects were comfortably seated in an adjustable armchair with a headrest. The left M1 target coordinates were individually localized in each subject based on hand knob location in T1-weighted image. This target position was marked on the subject’s anatomical MRI scan using Brainsight® (Rogue Research Inc., Montreal Quebec, Canada) software. The subject’s anatomical MRI scan was then co-registered with the subject’s head using frameless stereotaxy^43^. The subject’s head position was assessed using the Polaris (Northern Digital, Waterloo, Canada) infra-red tracking system to measure the position of scalp landmarks (nasion, nose-tip, and intratragal notch of the left and right ears) visible on the subject’s MRI. The TMS coil was placed over the target brain area. The root mean square of difference between the co-registered anatomical landmarks estimated by the neuronavigation software was limited below 2 mm for each subject for improving accuracy. After anatomic co-registration, a figure-of-eight coil was placed tangentially to the scalp in an orientation inducing a posterior-anterior current perpendicular to the main course of the central sulcus. The individual coil positioning parameters were stored in the neuronavigation software.

### Resting motor threshold (RMT)

Single-pulse TMS was delivered using a biphasic Magstim stimulator (Magstim Rapid, MagStim Company, Whitland, Wales, UK). Motor evoked potential (MEP) amplitudes were measured from the right first dorsal interosseous (FDI) muscle using an Ag/AgCl surface electrodes (Tyco healthcare, Neustadt, Germany) placed over the muscle belly (active) and metacarpophalangeal joint of the index finger. The electromyographic (EMG) signals were amplified and filtered with a 0.5-Hz high pass and 30–300 Hz band-pass using Synergy® instrument (Oxford Medelec, Wiesbaden, Germany). The RMT was defined as the lowest stimulus intensity eliciting at least five compound muscle action potentials (CMAP) amplitudes higher than 50 μV in 10 consecutive stimuli given over the motor hot spot^44^,^45^.

### TMS protocol

Repetitive TMS was administrated over the left M1 using a MagStim rapid stimulator with a hand-held figure-of-eight coil (70 mm standard coil, Magstim Rapid, MagStim Company, Whitland, UK). Stimulation intensity was set at 90% of the RMT of the right FDI muscle. A 90% RMT intensity was chosen because this is above the threshold for activating corticospinal output projections without central processing via sensory afferents.

In each rTMS session, 600 biphasic stimuli were given over the left precentral gyrus (M1) hand area. All subjects received 10-minute trains of 1-Hz rTMS delivered outside the fMRI scanner. We continuously monitored hand muscle twitching during stimulation. Control stimulation was delivered over the left M1 using the same stimulator output intensity. To reduce possible cortical stimulation effects in the control condition, the coil was angled at 45°, touching the skull with the rim opposite the handle^46^,^47^. The other stimulation parameters were similar to what has been described by Cardenas-Morales *et al*.^48^. Active and control rTMS sessions were counterbalanced. Participants were not asked to guess which session was active and which was control.

### fMRI

Subjects underwent fMRI while performing a simple finger-tapping task at approximately 1 Hz. MRI was performed with a General Electric Discovery 750w 3.0T MR scanner (GE Healthcare, Milwaukee, WI, USA), using a 24-channel head coil. fMRI parameters were as follows: T2* weighted echo-planar imaging sequences, repetition time (TR) = 3000 ms, echo time (TE) = 30 ms, flip angle (FA) = 90, matrix = 64 × 64, field of view (FOV) = 210 mm, slice thickness = 4 mm, no gap, and number of slice = 28–33. Scan time was approximately 3 minutes. The first four functional images of each run were discarded to minimize the effects of transient magnetic saturation. A three-dimensional (3-D) T1-weighted scan was obtained for structural reference. T1-weighted image parameters were as follows: 3-D fast spoiled gradient recalled (FSPGR) sequence, TR = 7.8 ms, TE = 3 ms, FA = 20, Inversion time = 450 ms, matrix = 256 × 256, FOV = 260 mm, slice thickness = 1.3 mm, no gap, and number of slice = 108.

### Image analysis

Image processing and statistical analyses were performed using MATLAB (The Math works Inc., Natick, MA, USA) and SPM8 (SPM; Wellcome Department of Imaging Neuroscience, London, UK; http://www.fil.ion.ucl.ac.uk). fMRI data were preprocessed using a sequential slice timing correction and spatially realigned to the first image to control for head movement and distortion. The realigned images were normalized to the Montreal Neurological Institute (MNI) brain. Normalized images were smoothed with the full-width at half maximum of the isotropic Gaussian kernel in 8 mm.

In first-level analysis, contrast images were obtained for each subject using the general linear model, contrasting each block of trials (motor finger-tapping task versus rest). The contrast images were entered into second-level analysis for group comparisons. A one-sample *t*-test was used to calculate inter-subject variability within the group. Statistical maps were corrected for multiple comparisons using false discovery rate (FDR) correction, and set at a threshold of p < 0.05 and minimum 64 contiguous voxels. One-way ANOVA (within subject) was used to assess differences in activationamong the four conditions (pre, post1, post2, and post3). The ANOVA had a threshold of p < 0.05 for 64 contiguous voxels and FDR-corrected for multiple comparisons across the whole brain. Post-hoc two sample t-tests were performed to determine differences between two conditions (pre > post1, pre < post1, pre > post2, pre < post2, pre > post3, pre < post3, post1 > post2, post1 < post2, post1 > post3, post1 < post3, post2 > post3, and post2 < post3). Post-hoc two sample t-tests had a threshold of p < 0.05 for 64 contiguous voxels with FDR-correction for multiple comparisons at the whole brain level. A Bonferroni correction was used to adjust for Type I errors at an alpha of 0.0084.

### Time correlation analysis

The individual time courses of seed points in each condition were obtained using SPM8. All seed points were selected on the basis of activation region in ANOVA analysis. Seed points included the bilateral precentral gyrus (M1), bilateral supplemental motor area (SMA), bilateral postcentral gyrus (S1), and bilateral inferior prefrontal region. The time course of each seed point was averaged for all subjects to calculate time course correlations between pre- and post-rTMS. The Pearson’s correlation coefficient was used to evaluate the relationship strength between time courses. The Pearson’s correlation coefficients were calculated for each seed point to show significant statistical differences at a p-value < 0.01. SPSS software (SPSS, Inc., Chicago, IL, USA, version 18) was used for all statistical analysis.

## Additional Information

**How to cite this article**: Min, Y.-S. *et al*. Neuromodulatory effects of offline low-frequency repetitive transcranial magnetic stimulation of the motor cortex: A functional magnetic resonance imaging study. *Sci. Rep*. **6**, 36058; doi: 10.1038/srep36058 (2016).

**Publisher’s note:** Springer Nature remains neutral with regard to jurisdictional claims in published maps and institutional affiliations.

## Supplementary Material

Supplementary Information
