# Supplementary material for: Neuromodulatory effects of offline low-frequency repetitive transcranial magnetic stimulation of the motor cortex: A functional magnetic resonance imaging study
Source: Sci Rep. 2016 Oct 27;6:36058. doi: 10.1038/srep36058 (PMC5081540; doi:10.1038/srep36058)
Supplement: Supplementary Information [file srep36058-s1.pdf]

Supplementary Information

Neuromodulatory effects of offline low-frequency repetitive transcranial magnetic stimulation of the motor cortex: A functional magnetic resonance imaging study

Yu-Sun Min, MD[1,2,+], Jang Woo Park, MS[3, +], Seong Uk Jin, MS[3], Kyung Eun Jang, MS[3], Byung-Joo Lee, MD[1], Hui Joong Lee, MD[4], Jongmin Lee, MD[4], Yang-Soo Lee, MD[1], Yongmin Chang, PhD[4,5,*] , Tae- Du Jung, MD[1,*]

Supplementary Table 1. MNI normalized left M1 coordinates for each subject

Subjects	MNI-coordinates		
	x	y	z
1	-36	-6.78	52.19
2	-34.01	-3.71	56.25
3	-36.97	-25	59.01
4	-30.02	-9.6	60.99
5	-37.33	-15.65	57.88
6	-37.97	-10.56	56
7	-32.04	-6.78	62.94
8	-46.49	-11.15	46.54
9	-37.4	-11.92	62.12
10	-29.47	-8.51	57.81
11	-34.59	-18.66	51.32
12	-25.22	-22.25	64.55
13	-21.73	-16.24	53.79
14	-38.71	-21.28	53.82
15	-46.34	-12.95	52.48
16	-42.36	-15.54	49.66
17	-36.16	-27.78	51.85
18	-43.93	-1.16	48.56
19	-37.4	-10.92	53.3

20	-38.16	-9.11	56.6

Supplementary Table 2. One-way ANOVA (within subject) result (FDR corrected for multiple comparison, P<0.05 and minimum cluster size of 64)

Brain region	Side	Cluster size	MNI-coordinates			Peak F
			x	y	z	
Postcentral Gyrus (S1)	L	658	-36	-36	42	10.02
	R	419	54	-26	46	8.03
Supplementary motor area (SMA)	L	205	2	12	46	9.26
	R	396	4	12	46	9.56
Inferior Parietal lobule	L	1376	-32	-46	54	10.58
	R	495	52	-30	50	7.84
Superior Parietal lobule	L	274	-30	-46	56	10.12
	R					
Inferior Frontal Gyrus	L	234	-54	8	6	9.60
	R	1037	60	14	6	14.21
Insula	L	84	-44	14	-10	7.93
	R	192	28	20	-20	7.46
Striatum	L					
	R	141	28	6	6	7.30
Inferior Temporal Gyrus	L	69	-48	-58	-6	8.11
	R					
Superior Temporal Gyrus	L	412	-44	18	-14	9.34

R	317	44	24	-20	9.68

Supplementary Table 3. Post-hoc two sample result between pre-stimulation and post 2 (FDR corrected for multiple comparison, P<0.05using a Bonferroni adjusted alpha level of 0.0084 and minimum cluster size of 64)

Brain region	Side	Cluster size	MNI-coordinates			Peak T
			x	y	z	
Activation (pre) > Deactivation (post2)						
Postcentral Gyrus (S1)	L	918	-36	-36	44	5.41
Supplementary motor area	L	484	-2	10	46	5.16
	R	704	4	10	46	5.22
Inferior Parietal lobule	L	931	-50	-28	38	5.41
	R	77	62	-22	20	4.52
Insula	L	217	-38	-4	10	4.13
	R	287	38	4	6	4.13
Striatum	L	71	-20	4	-2	3.47
	R	206	20	6	-2	4.01
Superior temporal gyrus	L	252	-62	-28	18	4.53
	R					
Activation (pre) < Deactivation (post2)						
Postcentral Gyrus (S1)	R	732	54	-26	44	4.86

Superior Parietal lobule	L	340	-30	-48	56	5.24
	R	172	30	-52	56	4.41
Inferior Frontal Gyrus	L	432	-42	20	-14	5.14
	R	636	58	18	4	5.49
Superior Frontal Gyrus	R	190	20	0	70	4.18
Middle Temporal Gyrus	L	825	-54	-12	-12	4.13
	R	497	66	-36	6	4.03
Cerebellum	L	106	-20	-46	-18	4.12

Supplementary Table 4. Time course correlation coefficient

Brain region (Coordinate)	Pre	Post1	Post2	Post3
Left motor cortex (M1)	1	0.968^{**}	0.979^{**}	0.976^{**}
Right motor cortex (M1)	1	0.940^{**}	0.940^{**}	0.940^{**}
Left SMA	1	0.884^{**}	-0.870^{**}	0.690^{**}
Right SMA	1	0.835^{**}	-0.908^{**}	-0.323^{*}
Left sensory cortex (S1)	1	0.943^{**}	-0.656^{**}	-0.882^{**}
Right sensory cortex (S1)	1	-0.614^{**}	-0.922^{**}	-0.897^{**}
Left inferior frontal cortex	1	0.899^{**}	-0.485^{**}	0.922^{**}
Right inferior frontal cortex	1	0.897^{**}	0.384^{**}	0.562^{**}

** $p < 0.01$, * $p < 0.05$

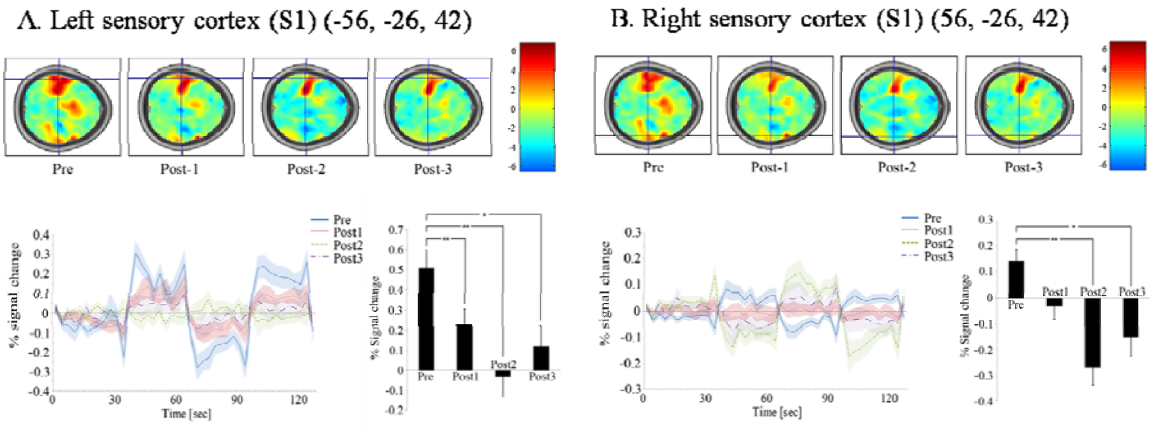

Supplementary Figure 1. Results of the right finger-tapping task during four BOLD fMRI scans before and after 1-Hz rTMS of the left (A) and right sensory cortex (B).Group one sample t-test maps had no threshold to show time course of changes in whole brain neural activity. (A) A time course of BOLD activity in the left S1 showed changes from positive responses (activation) from pre-stimulation to slightly negative responses (deactivation) at 20 minutes after rTMS (post2), then back to positive responses (activation). (B) Time course of BOLD activity in the right S1 showed changes from positive responses (activation) from pre-stimulation to negative responses (deactivation) at post1, post2 and post3. (*) P<0.05 and (**) P<0.01. Plots showed means (lines) and SEMs (shading) of percentage signal changes.

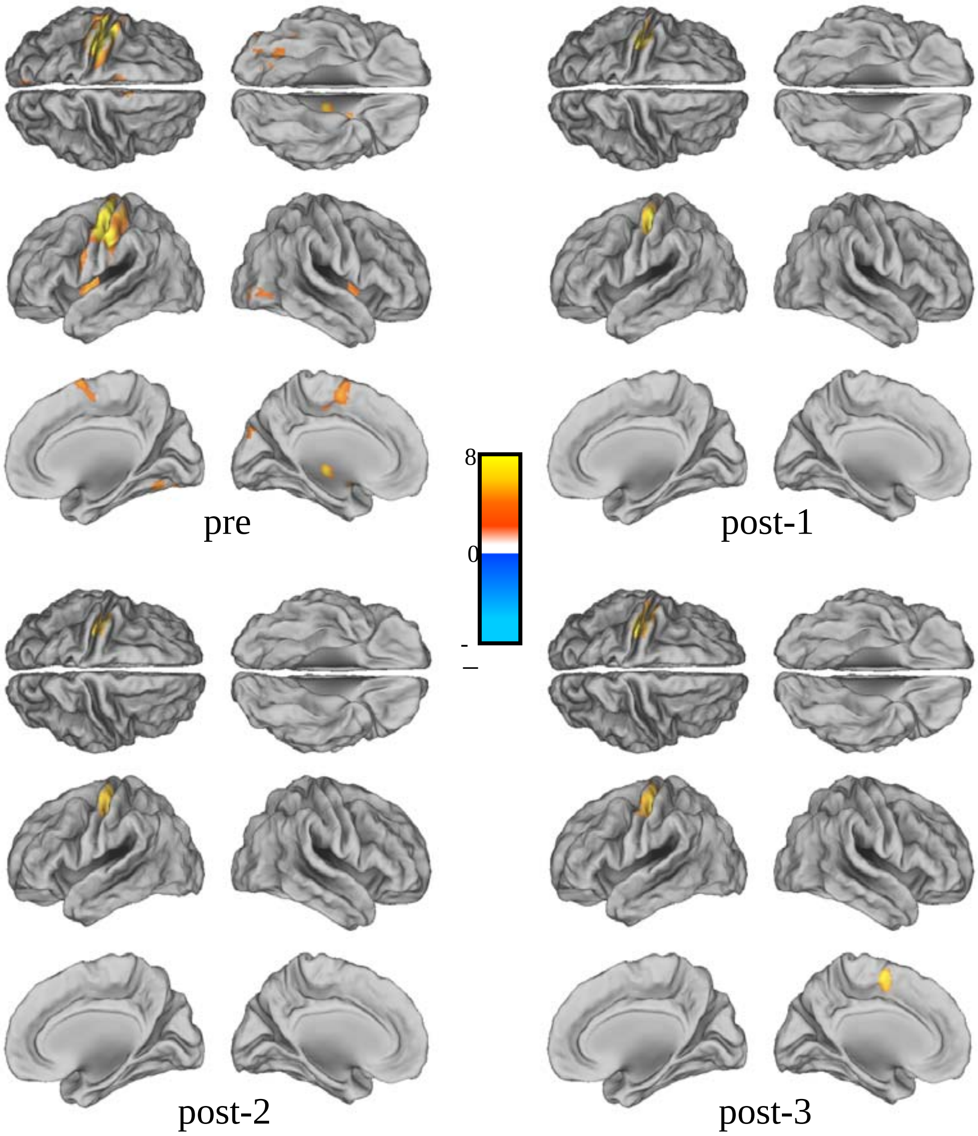

Supplementary Figure 2. Group one sample t-test results (p < 0.05, FDR-corrected for multiple comparisons at the whole brain level) during the finger-tapping task at pre- and three post-sham stimulation time points (post-1, post-2, post-3). Activation (motor task > rest) was presented in yellow.
